# Merge-and-Split Graph Convolutional Network for Skeleton-Based Interaction Recognition

**DOI:** 10.34133/cbsystems.0102

**Published:** 2024-03-20

**Authors:** Haoqiang Wang, Yong Wang, Sheng Yan, Xin Du, Yuan Gao, Hong Liu

**Affiliations:** ^1^ School of Artificial Intelligence, Chongqing University of Technology, Chongqing, China.; ^2^ Computing Sciences (CS), Faculty of Information Technology and Communication Sciences (ITC), Tampere University, Tampere, Finland.; ^3^ Key Laboratory of Machine Perception, Shenzhen Graduate School, Peking University, Beijing, China.

## Abstract

We introduce an innovative approach to address a significant challenge in interaction recognition, specifically the capture of correlation features between different interaction body parts. These features are often overlooked by traditional graph convolution networks commonly used in interaction recognition tasks. Our solution, the Merge-and-Split Graph Convolutional Network, takes a unique perspective, treating interaction recognition as a global problem. It leverages a Merge-and-Split Graph structure to effectively capture dependencies between interaction body parts. To extract the essential interaction features, we introduce the Merge-and-Split Graph Convolution module, which seamlessly combines the Merge-and-Split Graph with Graph Convolutional Networks. This fusion enables the extraction of rich semantic information between adjacent joint points. In addition, we introduce a Short-term Dependence module designed to extract joint and motion characteristics specific to each type of interaction. Furthermore, to extract correlation features between different hierarchical sets, we present the Hierarchical Guided Attention Module. This module plays a crucial role in highlighting the relevant hierarchical sets that contain essential interaction information. The effectiveness of our proposed model is demonstrated by achieving state-of-the-art performance on 2 widely recognized datasets, namely, the NTU60 and NTU120 interaction datasets. Our model’s efficacy is rigorously validated through extensive experiments, and we have made the code available for the research community at https://github.com/wanghq05/MS-GCN/.

## Introduction

As a popular research direction, human interaction recognition [1–4] has a wide reference prospect in the field of robotics, such as human–computer interaction [5–12]. Robots can pay attention to human interaction, identify human behaviors [13–16], and also identify behaviors between robots. In recent years, with the continuous development of deep learning, interaction recognition based on RGB [17–19] and skeleton-based [20–26] has been proposed. However, the RGB-based method is affected by illumination, self-occlusion, and other factors, so it cannot effectively carry out interaction recognition, while the skeleton-based interaction recognition method is not affected by these factors and has achieved remarkable results.

**Fig. 1.**
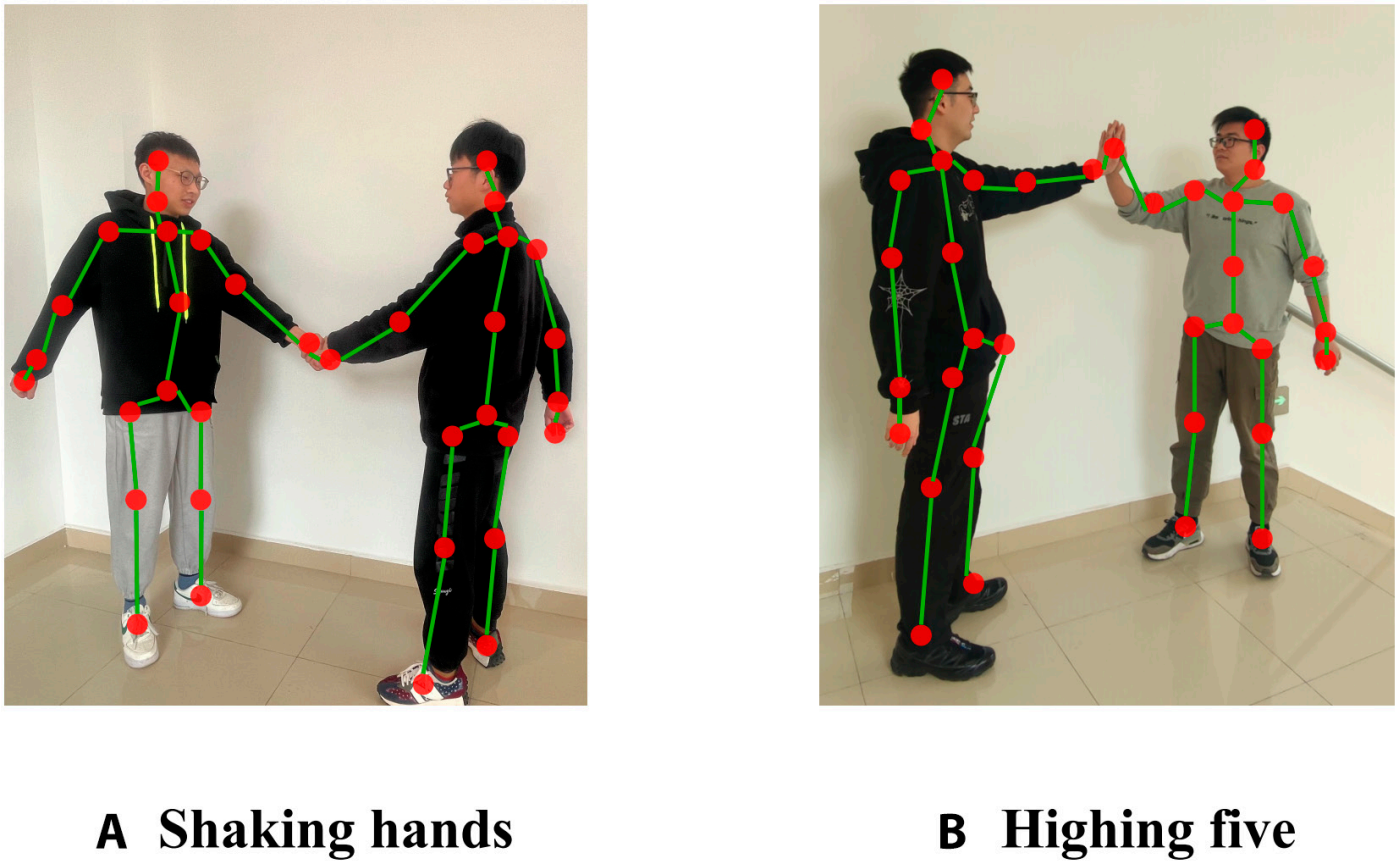
The interaction action of "shaking hands" and "highing five". In both actions, "shaking hands" (A) and "highing five" (B), there is a semantic correlation between the hand and the hand, and their subtle difference lies in the evolution of the distance.

Traditional skeleton-based interaction recognition methods [3,27–34] consider human joints as independent features and construct feature sequences from them for input to recurrent neural network or convolutional neural network for action prediction. However, these methods overlook the correlation features between cross-joints. As shown in Fig. 1, the feature information between interactive behaviors is aggregated at the interaction part, and it is difficult to extract their interaction information and distinguish the subtle differences between similar actions of “handshake” and “high five”. Meanwhile, Perez et al. [35] proposed an LSTM-based 2-stream interaction relationship network, referred to as LSTM-IRN, to model the internal relationship between the body joints of the same person and the relationship between the joints of different people. However, LSTM-IRN did not consider the distance relationship between body parts and was unable to learn relevant features between the 2 interaction parts.

Most of the existing single-person action recognition methods [3,36–43] have embraced the concept of graph convolution. However, in action recognition, we often encounter scenarios involving both dual-person interactions and single-person movements. Traditional graph convolution models tend to handle dual-person interactions by treating the 2 individuals separately as isolated entities, thus overlooking the crucial interaction information between them.

Therefore, in order to effectively extract the interaction information between behaviors, we propose a Merge-and-Split Graph Convolutional Network (MS-GCN), including Merge-and-Split Graph Convolution (MSGC) combining GCN and Merge-and-Split Graph, a Short-term Dependence (STD) module, and a Hierarchical Guided Attention (HGA) module.

MSGC combines GCN with our Merge-and-Split graph, which merges the node information of 2 individuals in the same feature space, and maps the nodes of corresponding hierarchical sets of the 2 individuals in the same semantic space. We start by choosing the abdominal node as the initial node and expanding it outward to form the same semantic space. A set of nodes sharing the same semantic space is defined as a hierarchical set. It can be seen that in the interaction behavior, the feature information is concentrated among the interaction parts. Therefore, when we carry out interaction identification, we need to focus on learning the relevant features of the same hierarchical set of interaction nodes.

In general, temporal convolution tends to focus more on the long-term dependence of human motion and less on short-term variations in human motion. However, short-term changes are often important to identify subtle differences between different actions. Therefore, we propose a STD. STD directly conducts a Short-term Dependence module on the original skeleton data, enabling subsequent convolutional networks to simultaneously extract and learn joint information and motion information to better identify similar motions.

Among different interaction behaviors, we need to pay attention to different hierarchical sets, such as the “hand waving” action, and we need to pay more attention to the hierarchical sets with node data of the hand. Therefore, we propose a HGA module, extract node features of each hierarchical set, and select hierarchical sets with a relatively close distance between the hierarchical sets of interaction behaviors by calculating Euclidean distance.

The main contributions of this paper are as follows:

• We propose the Merge-and-Spilt Graph Convolutional Network, which can effectively extract interaction information compared to other interaction recognition methods.

• We propose the STD module, MSGC, and HGA module to extract the correlation features among interaction behaviors. MSGC integrates a fused split graph by merging 2-person node information into the same semantic space and splitting them into hierarchical sets to extract features within the same semantic space. STD extracts spatial and motion features of interaction behaviors, while HGA highlights the hierarchical features with higher correlations among interaction behaviors.

• Our MS-GCN achieved state-of-the-art results on the NTU-RGB+D and NTU-RGB+D 120 interaction datasets, and we also demonstrated good performance on single-person action recognition based on skeletal data.

## Related Works

### Action recognition with GCN

In skeleton-based action recognition, the human skeleton is constructed as a graph structure with joint nodes [38,39,44–49]. To deal with non-Euclidean data such as graphs, graph convolution has been proposed. Yan et al. [23] proposed ST-GCN, which first uses topological maps to extract and aggregate the spatiotemporal information of bones. However, ST-GCN uses handcrafted skeleton graphs and cannot extract correlation features between joints that are not naturally connected. Chen et al. [39] further proposed the CTR-GCN to simultaneously learn the shared topology and channel correlation, learn the unnatural connections between joint nodes, and enhance the feature extraction and aggregation capabilities of GCN without significantly increasing the parameters. Liu et al. [24] proposed a Mask-GCN to focus on learning action-specific skeleton joints that mainly convey action information meanwhile masking action-agnostic skeleton joints that convey rare action information and suffer more from novel motion patterns to learn stable and information-rich motion features. In this work, we focus on skeleton-based interaction recognition and propose to model human interactions from both semantic and motion information levels.

### Human interaction recognition

With the development of deep learning, graph convolutional neural networks (GCNs) have been widely used in skeleton-based action recognition and interaction recognition. For example, Gao et al. [50] proposed Attentional Interaction Graph Convolutional Networks (AIGCN) to explore 2-person interactions. An Interactive Attention encoding GCN (IAE-GCN) module is designed to extract the interactive spatial structure, and the joint position encoding is treated as semantic information to reflect their influence on each other. Secondly, the interactive attention mask TCN (IAM-TCN) is used to extract the time interaction features, representing the time attention incentive signals between different joints of different people. LI et al. [51] proposed 2P-GCN, introduced a new unified 2-person graph to represent interbody and intrabody correlations between joints, and designed several graph labeling strategies to supervise the model’s learning to distinguish spatiotemporal interaction features. Some studies have utilized pose estimation and keypoint detection techniques for extracting features of human interaction behaviors, besides using GCNs. For example, Chen et al. [3] proposed a behavior recognition method based on pose estimation that first extracts human poses using convolutional neural networks and then models the time series using LSTM to obtain behavior representation. In addition, Liu et al. [33] presented an interaction behavior modeling approach based on pose estimation and keypoint detection that employs a 2-stage pipeline consisting of pose estimation and keypoint detection networks for extracting features of interaction behaviors. These methods overlook the correlated features between the interactions of 2 individuals. In contrast, we fuse the interactions of 2 individuals into holistic interaction recognition, where we extract their distance and semantic information at the global level to capture their interactive features.

### Attention mechanism for action recognition

The attention mechanism [9] was first proposed in natural language processing, which is the basic element of deep neural networks. More recently, attention networks such as Hu et al. [41] proposed SEnet to introduce attention mechanisms into computer vision. SEnet networks use spatially oriented attention and multi-layer perceptrons to extract channel information. Woo et al. [34] further propose CBAM, which uses channel-oriented and spatial-oriented attention, inputs an intermediate feature map, and the CBAM module infers the attention map along the 2 independent dimensions (channel and space), then multiplies the attention map with the input feature map for adaptive feature optimization. In the context of GCNs, attention mechanisms have been proposed to sum the features of neighboring nodes [42,43]. The weight of adjacent node features is determined solely by the node features, independent of the underlying graph structure. In this work, we propose HGA, highlighting the hierarchical features that are more relevant in the interaction behavior, and learning subtle differences between interactions.

## Merge-and-Split Graph Convolutional Network

**Fig. 2.**
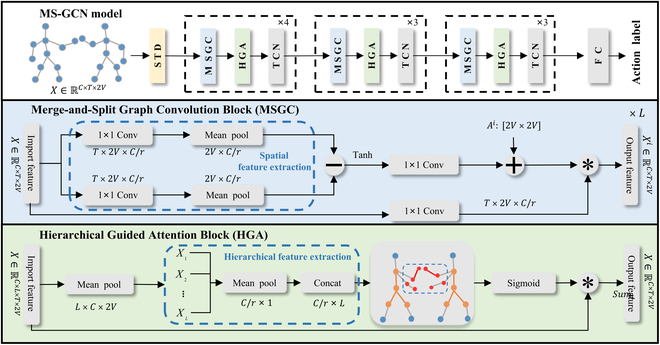
The overall architecture of the MS-GCN. The interaction skeleton data is first fed into the STD module to extract short-term temporal features. The MS-GCN mainly aggregates feature information among joint nodes and then feeds it into the Hierarchical Graph Attenion (HGA) module to highlight the features of the main hierarchical set. The input features go through MSGC modules to extract the feature information for each hierarchical set. In the HGA module, we assign adaptive weights to the input features and then sum them along the hierarchical dimension. In the TCN module, different durations are modeled.

As shown in Fig. 2, our MS-GCN contains STD modules, MSGC and HGA modules. STD extracts spatial and motion features of interaction behaviors, MSGC extracts node features in each hierarchical set and aggregates all hierarchical outputs, and HGA highlights the hierarchical sets with greater relevance in the interaction behavior. We also propose a new graph structure, the Merge-and-Split Graph for interaction recognition. To model actions with different durations, we use a multiscale temporal modeling module following (TCN)[39]. The proposed model is effective not only for interaction recognition but also for individual action recognition.

### Merge-and-Split Graph

**Fig. 3.**
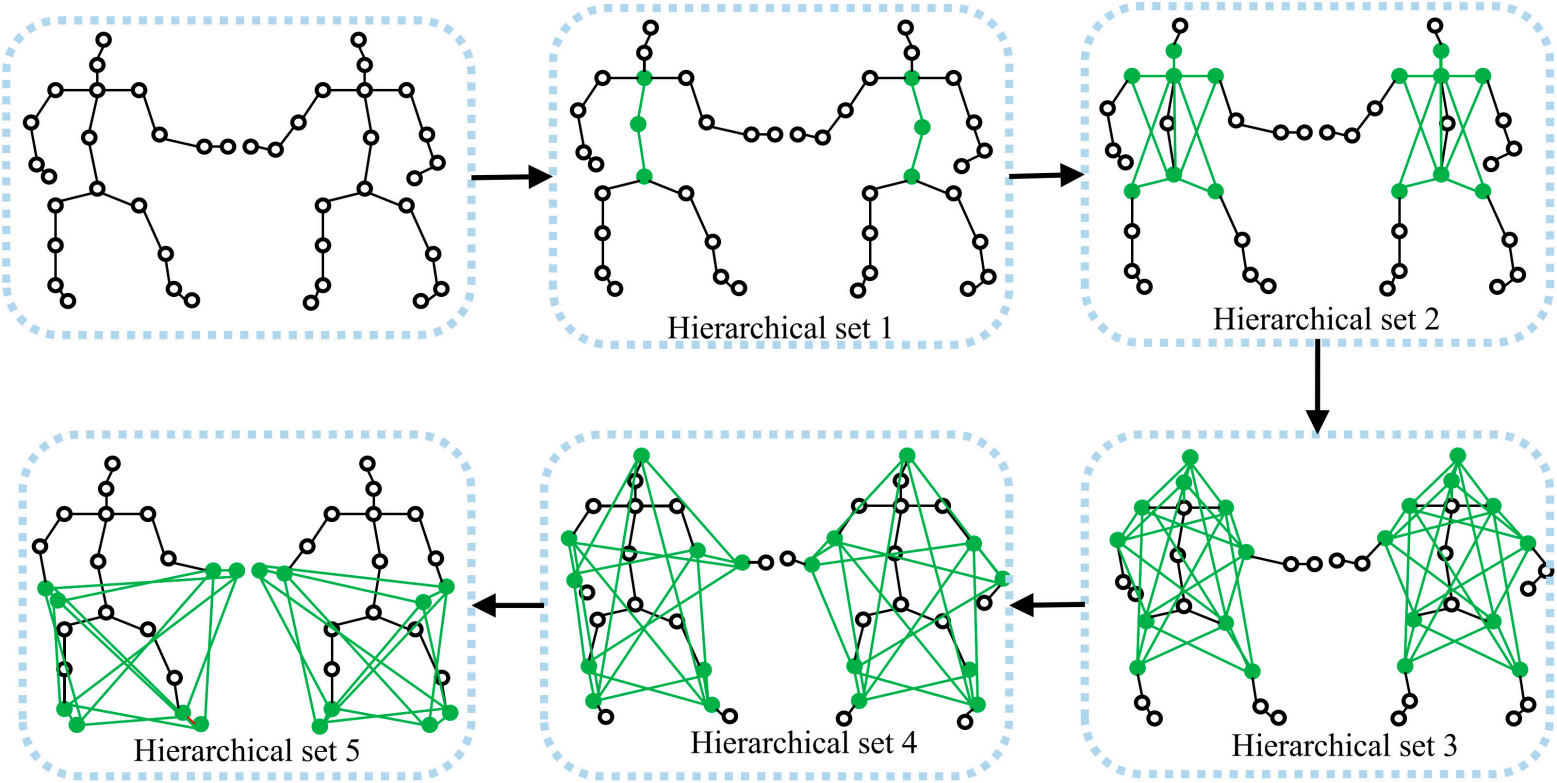
Merge-and-Split Graph. The 2-person skeleton data are merged in the same semantic space and split into different hierarchical sets, where green lines and green nodes represent edges and nodes of the corresponding hierarchical sets.

The traditional human skeleton graph construction method uses natural connections as edges and lacks information on the connections between the joint nodes and the border nodes. The Merge-and-Split Graph proposed in this paper is the latest human skeleton graph construction method, which merges the 2-person joint nodes and splits them into multiple hierarchical sets in the semantic space. Figure 3 gives the framework for Merge-and-Split Graphs.

The construction of the Merge-and-Split graph involves 2 key steps. First, we merge the key points of 2 individuals into a unified whole. The number of human nodes in the NTU dataset is 25 nodes, and we combine the nodes of 2 people to form a 2-person overall graph of 50 nodes. This overall graph shares the same feature space. Subsequently, by selecting the starting points whose adjacent nodes exist in the same semantic space, we divide the entire graph into different hierarchical sets. We choose the abdominal node as the starting point and decompose the graph sequentially through the abdominal node. This allows nodes concentrated in the same hierarchical structure to exist in the same semantic space. As shown in Fig. 3, the abdominal node and its adjacent nodes are placed in the same hierarchical set. Adjacent nodes share the same hierarchical set with their corresponding nodes, and this process is repeated to construct 6 hierarchical sets. The hierarchical sets contain key points with related connectivity information, forming different semantic spaces, enabling more effective and accurate analysis of joint movements. Hierarchical node sets are formed by grouping nodes in the same semantic space, such as hand joints and foot joints of 2 people. To construct the graph, adjacent hierarchical node sets are sequentially connected with edges that represent the connection relationship between nodes in adjacent sets. The resulting Merge-and-Split graph can effectively extract features that capture joint interactions and evolve characteristics of distance and joint movements. After connecting sequentially, the constructed graph structure contains the hierarchical information of the graph and defines ***A***_***i***_ ∈ ℝ^*C*×2*V*×2*V*^ as the adjacency matrix of the adjacent hierarchical set and ***A*** ∈ ℝ^*C*×*L*×2*V*×2*V*^ as the adjacency matrix of the whole graph structure. It is worth noting that 2*V* represents the number of joint nodes for 2 persons, *C* represents the channel dimension of the adjacency matrix and *L* represents the number of hierarchical sets. Mathematically, this process is formulated as

$$A_{i}=\theta_{i}\left(H_{i}\to H_{i+1}\left\|H_{i+1}\to H_{i}\right\|H_{i}+H_{i+1}\right)$$ (1)

where *H_i_* represents the *i*-th hierarchical set of nodes and the *H_i_* → *H*_*i*+1_ denotes a set of edges form *H_i_* to *H*_*i*+1_. Note that ‖ is a cascade of operations. We normalize *θ_i_* the adjacency matrix to prevent the inner product of the adjacency matrix and the feature matrix from changing the original feature distribution. The adjacency matrix of the graph formed by the *i*-th hierarchical sets and *i*+1 hierarchical sets is defined as ***A***, which does not contain the connection information of the other hierarchical sets.

In order to take into account the information of the entire global graph, the edge connections between each hierarchical set must be taken into account, so the adjacency matrix ***A*** is formulated as

$$A=\left[A_{1}\left\|A_{2}\right\|A_{3}\ldots ∥A_{L}\right]$$ (2)

where *L* represents the number of our hierarchical sets. Through Merge-and-Spilt, we construct adjacency matrices ***A*** ∈ ℝ^*C*×*L*×2*V*×2*V*^ with different semantic spatial information.

### Short-term Dependence

**Fig. 4.**
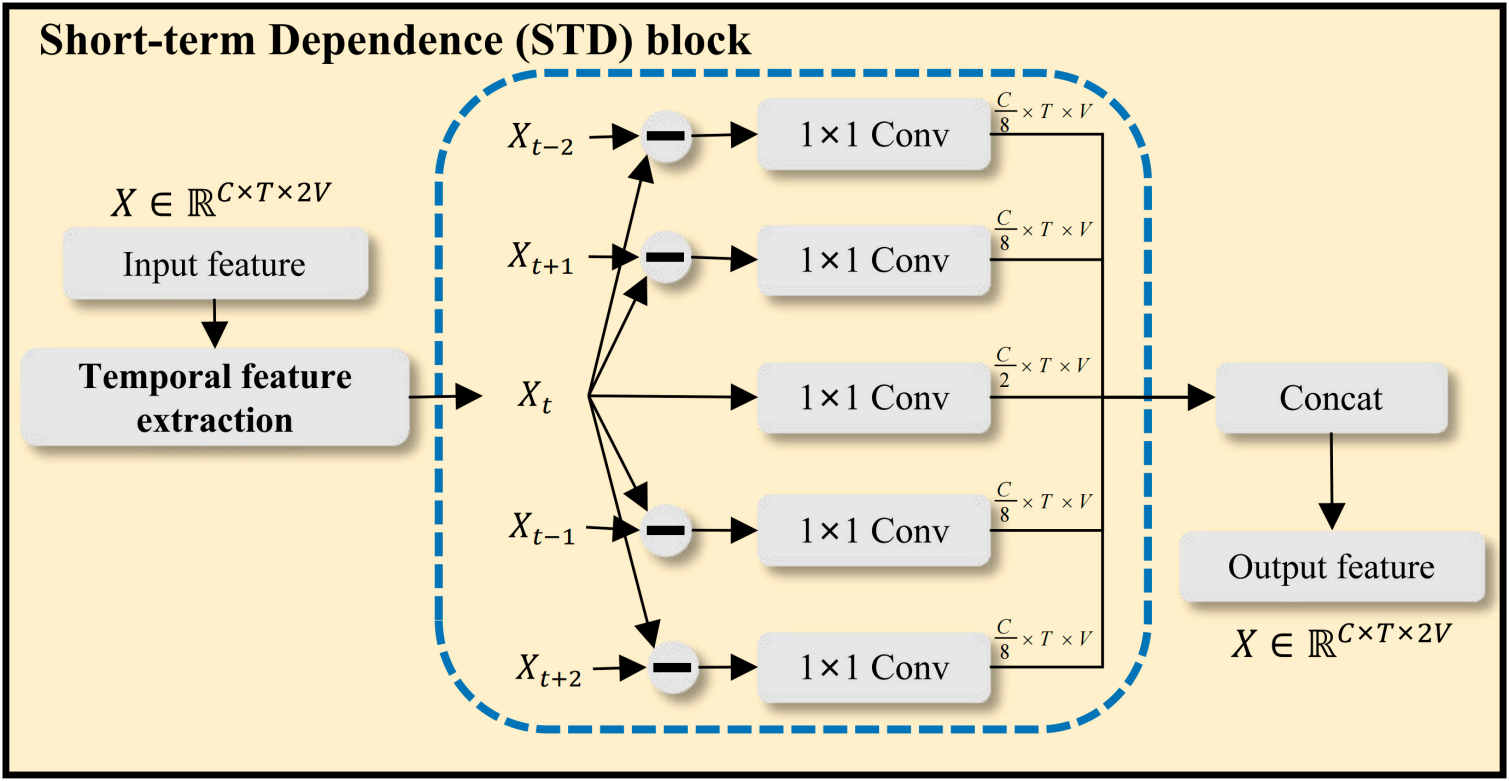
STD block. Temporal feature extraction is the operation to extract spatiotemporal information, and - is matrix subtraction.

The traditional interaction recognition based on GCNs generally focuses on the long-term dependence of behavior, ignores the key information of time and space, and lacks attention to the short-term changes of behavior. However, short-term changes in time and space are also important for recognizing human behavior and extracting short-term changes in motion makes it easier to learn subtle differences between similar movements. Therefore, as shown in Fig. 4, we propose an STD module to model short-term motion information and joint features in temporal space.

To extract the short-term motion information in the spatiotemporal, we first make 4 copies of the input ***X*** ∈ ℝ^*C*×*T*×2*V*^ and subtract 1 or 2 frames of temporal information *T* from the start or end of the motion. Their dimension can be expressed as ***X***_***t*** − **2**_ ∈ ℝ^*C*×[0:*T*−2]×2*V*^, ***X***_***t***−**1**_ ∈ ℝ^*C*×[0:*T*−1]×2*V*^, ***X***_***t***+**1**_ ∈ ℝ^*C*×[1:*T*]×2*V*^, ***X***_***t***+**2**_ ∈ ℝ^*C*×[2:*T*]×2*V*^. After extracting temporal frames, the motion termination time is padded with zeros, and the difference is computed to extract short-term motion information. As illustrated in Fig. 4, the extracted information is concatenated with the original motion information, incorporating the short-term motion information into the original feature space. By concatenating the input features and short-term motion features, joint information and short-term motion information are obtained, resulting in the output *Z* as follows.

$$Z=\left[\left(]X-X_{t-1}\right)\left\|\left(X-X_{t+1}\right)\ldots \right\|X\right]$$ (3)

where ***X***_***t***−**2**_, ***X***_***t***−**1**_, ***X***_***t***+**1**_ ***X***_***t***+**2**_ is the copy that we created. *conv* is a feature transformation of short-term motion features. We subtract the copies in turn from the input copies, and the new features generated contain the short-term motion features. Concatenate ∥ the short motion feature with the input feature ***X*** ∈ ℝ^*C*×*T*×2*V*^. Therefore, the output ***Z*** contains the joint information and short-term motion information.

### Merge-and-Split Graph Convolution

Our MSGC is shown in Fig. 2. First, we use a linear transformation to transform the input feature ***X*** ∈ ℝ^*C*×*T*×2*V*^ into high-level features. Advanced spatial features are extracted by reducing the factor “r”. To prevent data redundancy, we average the temporal features and focus on the spatial features, and then we use the correlation modeling function $M\left(\cdot \right)$ to extract the spatial distribution features between each joint of the 2-person joint under different motions and use *φ* and *ω* to reduce the feature dimension. We add the hierarchical adjacency matrix ***A***_***i***_ ∈ ℝ^*C*×2*V*×2*V*^ to the joint features to obtain the pairwise correlation between the joint nodes (***x***_***i***_, ***x***_***j***_). It is worth noting that the adjacency matrix ***A*** ∈ ℝ^*C*×*L*×2*V*×2*V*^ is a learnable parameter, and unlike other GCNs, we put the connection of edges in one dimension. Finally, we perform feature aggregation for each channel and parallel the outputs of all hierarchical channels to finally obtain ***H***

$$M_{\mathrm{ij}}=\sigma \left(\varphi \left(x_{i}\right)-\omega \left(x_{j}\right)\right),i,j\in \left\{1,2\ldots 2V\right\}$$ (4)

$$H_{i}=X⊗\left(\alpha \cdot M\left(X\right)+A_{i}\right)$$ (5)

$$H=\left[H_{1}\left\|H_{2}\right\|H_{i}\ldots ∥H_{L}\right]$$ (6)

where *α* is a trainable scalar to adjust the strength of the refinement, preventing the adjacency matrix ***A*** from having too high a connection strength of edges, resulting in not learning subtle differences between the interaction behaviors. The input feature ***X*** ∈ ℝ^*C*×2*V*×2*V*^ is the transformed high-level feature. *σ*(·) is the activation function. The subtraction operation essentially computes the distance between *φ*(***x***_***i***_) and *ω*(***x***_***j***_) in the channel dimension and extracts the spatial information between the nodes. We use the correlation modeling function $M\left(\;\cdot \;\right)$ to capture the spatial distribution information of the connection points between channels. Then we add the spatial distribution feature between the joint nodes and the hierarchical adjacency matrix ***A***_***i***_ and add the edge connection information between the hierarchical node set to the feature. ⊗ is the operation of the Einstein summation convention. The input feature and each channel diagram are aggregated to obtain the output features ***H***_***i***_ ∈ ℝ^*C*×*T* ×2*V*^ of the single hierarchical set. We concatenate these output ***H***_***i***_ values to the channel dimension to obtain the final output ***H*** ∈ ℝ^*C*×*L*×*T*×2*V*^.

### Hierarchical Guided Attention

The MSGC uses an aggregation strategy to add all the hierarchically structured outputs. However, since the interaction behavior may be linked between the joint points and the Marginal joint nodes, we propose a HGA module, which applies a weighted sum strategy to the hierarchical dimensions and gives appropriate attention to the hierarchical output.

Our HGA module is applied on the feature map ***H*** ∈ ℝ^*C*×*L*×*T*×2*V*^ after the MSGC layer. To ensure a suitable attention score, we first extract representative nodes in each hierarchy set, as the number of edges connected to each node may vary significantly. This representative node extraction step is crucial in achieving accurate attention scores. Once extracted, the representative nodes are spatially pooled to further enhance the effectiveness of our approach. The spatial pooling function *∂* is formulated as

$$\partial \left(H^{\left(i\right)}\right)=\frac{1}{L_{i}+L_{i+1}}\;\sum_{v_{j}\in \left(L_{i}+L_{i+1}\right)}\left(\frac{1}{T}H^{\left(i\right)}\left(v\right)\right)$$ (7)

where *L_i_* denotes the number of joint nodes in the *i*-th hierarchical set and the *v_j_* is the representative node in the neighboring hierarchical set. We perform a pooling operation on it in the time dimension *T*.

When the hierarchical information of representative nodes is extracted, each hierarchical feature does not share information. We learn the similarity in the hierarchical feature space by Euclidean distance and multiply the feature maps with all hierarchical information by the MSGC output feature map to obtain the output feature map by the hierarchical dimension weighted sum. The output feature map ***A*** is formulated as

$$Z=\sum_{i=1}^{L}X\cdot \text{sigmoid}\;\left(\underset{i\in L}{∥}B\left(H^{\left(i\right)}\right)\right)$$ (8)

after concatenating the output ***H***^(***i***)^ of the MSGC hierarchical set into one dimension, containing the feature information of each hierarchical set. We learn the feature similarity between each hierarchical set by Euclidean distance B and splice the feature similarity of each hierarchical set on the level dimension (*i* ∈ *L*) after extracting. The hierarchy is passed through a sigmoid activation function, which assigns adaptive weights to the features of each hierarchical set and multiplies them with the input feature mapping to finally obtain the output feature ***Z*** ∈ ℝ^*C*×*T*×2*V*^.

## Experiments

### Datasets and settings

NTU-RGB+D(NTU-60) [52] dataset consists of 40,320 action clips from 60 action classes performed by 25 actors from 3 different viewpoints. It is currently one of the largest action recognition datasets based on a single depth sensor. Each sample in this dataset consists of 3D human joint position sequences recorded by a depth sensor. The actions in this dataset include common daily and sports actions, such as running, walking, and bending. The NTU-60 dataset contains 11 2-person interaction action categories, and we refer to this part of the dataset as NTU-60*.

NTU-RGB+D120(NTU-120) [53] dataset is an extension of the NTU-RGB+D dataset and contains 118,081 action clips from 120 action classes. Like the NTU-RGB+D dataset, it is also based on a single depth sensor and contains 3D human joint position sequences. However, the NTU-RGB+D120 dataset is more diverse and complex, with a wider range of human poses, actions, and environmental conditions. The NTU-120 dataset contains 26 2-person interaction action categories, and we refer to this part of the dataset as NTU-120*.

Our baseline model is CTR-GCN [39], which proposes a novel channel topology refinement graph convolution to dynamically learn different topologies and efficiently aggregate joint features in different channels for skeleton-based action recognition.

In our experiments, we set the batch size to 16 and used the data preprocessing method of CTR-GCN. Our model is trained with SGD with a momentum of 0.9 and weight decay of 0.0004. The training epoch is set to 65, and the warm-up strategy [54] is used for the first 5 epochs to make the training process more stable. The learning rate is dynamically attenuated [55], with a maximum of 0.1 and a minimum of 0.0001. All our experiments are carried out on the RTX5000. It needs 8 h to train the NTU120 dataset single joint modality. In traditional behavior recognition, it is common to integrate results from 4 modalities: joint, bone, joint motion, and bone motion. In this study, we conducted ablation experiments using the joint modality. The final experimental results are based on the fusion of the joint modality and the bone modality.

### Ablation study

In this section, we conduct extensive ablation studies on the NTU-120* dataset to verify the effectiveness of the proposed MSGC, STD module, and HGA module. In our ablation study, we use only joint modality data and adopt a cross-subject (X-Sub) scheme. The results of the ablation study are shown in Table 1.

**Table 1.** Ablation study of our proposed MS-GCN

Method	NTU-120* X-Sub (%)
baseline*_joint_*	87.67
+ Single Spilt Graph Convolution	88.32^↑0.65^
+ MSGC	88.68^↑1.01^
+ MSGC + STD	89.16^↑1.49^
+ MSGC + HGA	89.19^↑1.52^
+ MSGC + STD + HGA	89.59^↑1.92^

**Fig. 5.**
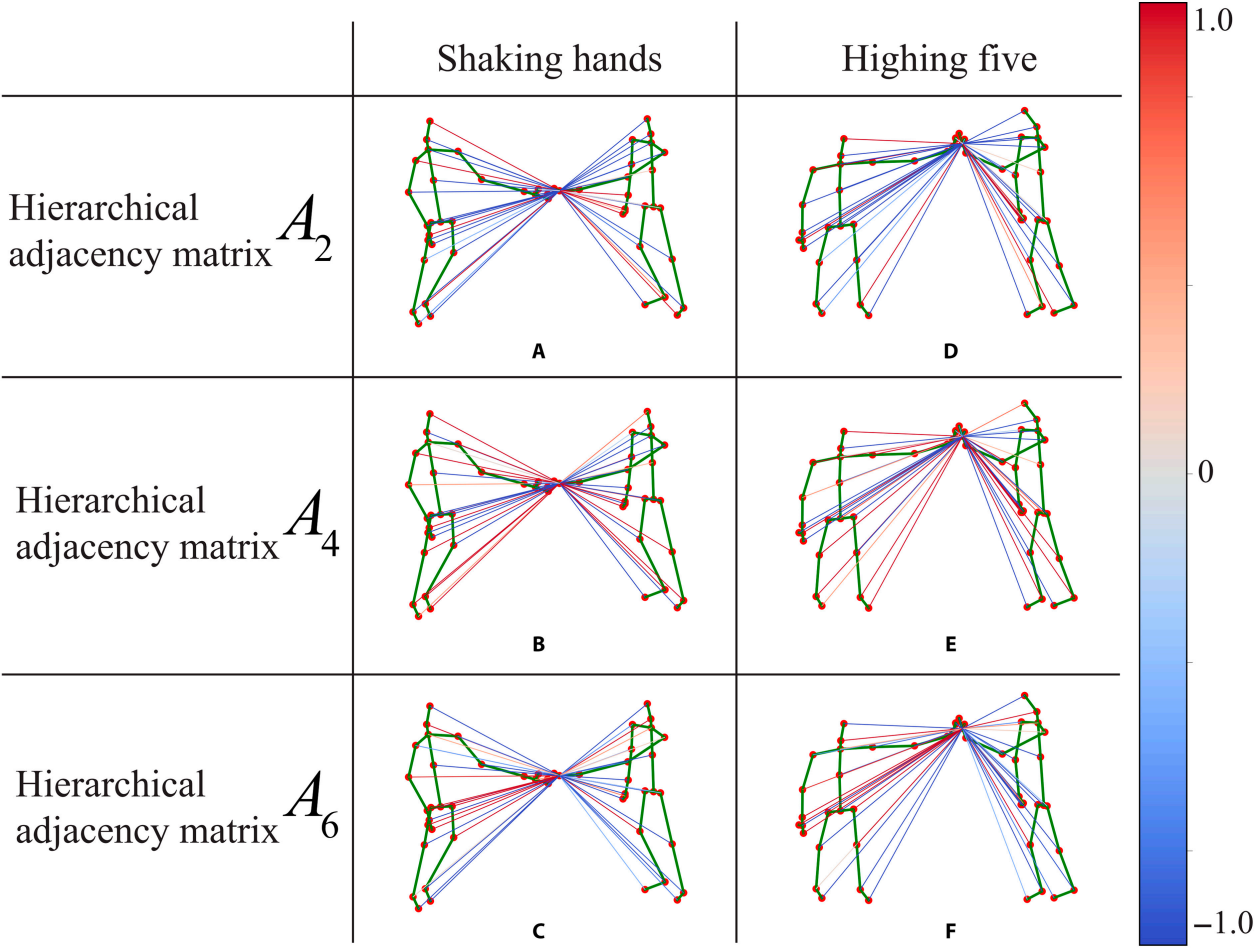
Visualization of the "shaking hands" and "highing five" actions. We select the hierarchical adjacency matrices of the second, fourth, and sixth hierarchical sets. (A) to (C) are the corresponding connections between the hand joints and all other double joints under the hierarchical adjacency matrix for the "shaking hands" action, and (D) to (F) correspond to the "highing five" action.

First, we verify the effectiveness of the MSGC. We treat the 2-person interaction as 2 single-people interactions and only use our splitting idea when constructing the graph. It can be found that our splitting idea is also effective when dealing with 2-person interaction, improving the accuracy by 0.65%. Compared to a single-splitting graph, our proposed Merge-and-Split graph achieves a 1.01% improvement in accuracy and 0.36% improvement compared to using a single-person split graph. It can be found that our Merge-and-Split idea is easier to extract interaction information than the single-splitting idea. The visualization result of our Merge-and-Split graph is shown in Fig. 5, and we set the adjacency matrix as a learnable parameter and choose the adjacency matrix of the second, fourth, and sixth hierarchical sets. For the “shaking hands” and “highing five” actions, the features are mainly clustered around the hand, reflecting the evolution of the distance and joint features during the movement. We choose the connections between the hand node and all other joints, and the shades of color represent the strength of the connections between the joints. The closer the value of color to zero indicates weaker connectivity between joints. There are obvious interactive features of the hand in the handshake action, and the relevant connections of the hand joint nodes are included in the fourth (Fig. 5B and E) and sixth (Fig. 5C and F) layer sets. Compared with Fig. 5A and D, the connection strength between our hand and the interaction parts is higher, indicating that our MS-GCN can learn the correlation features between the interaction joints.

**Fig. 6.**
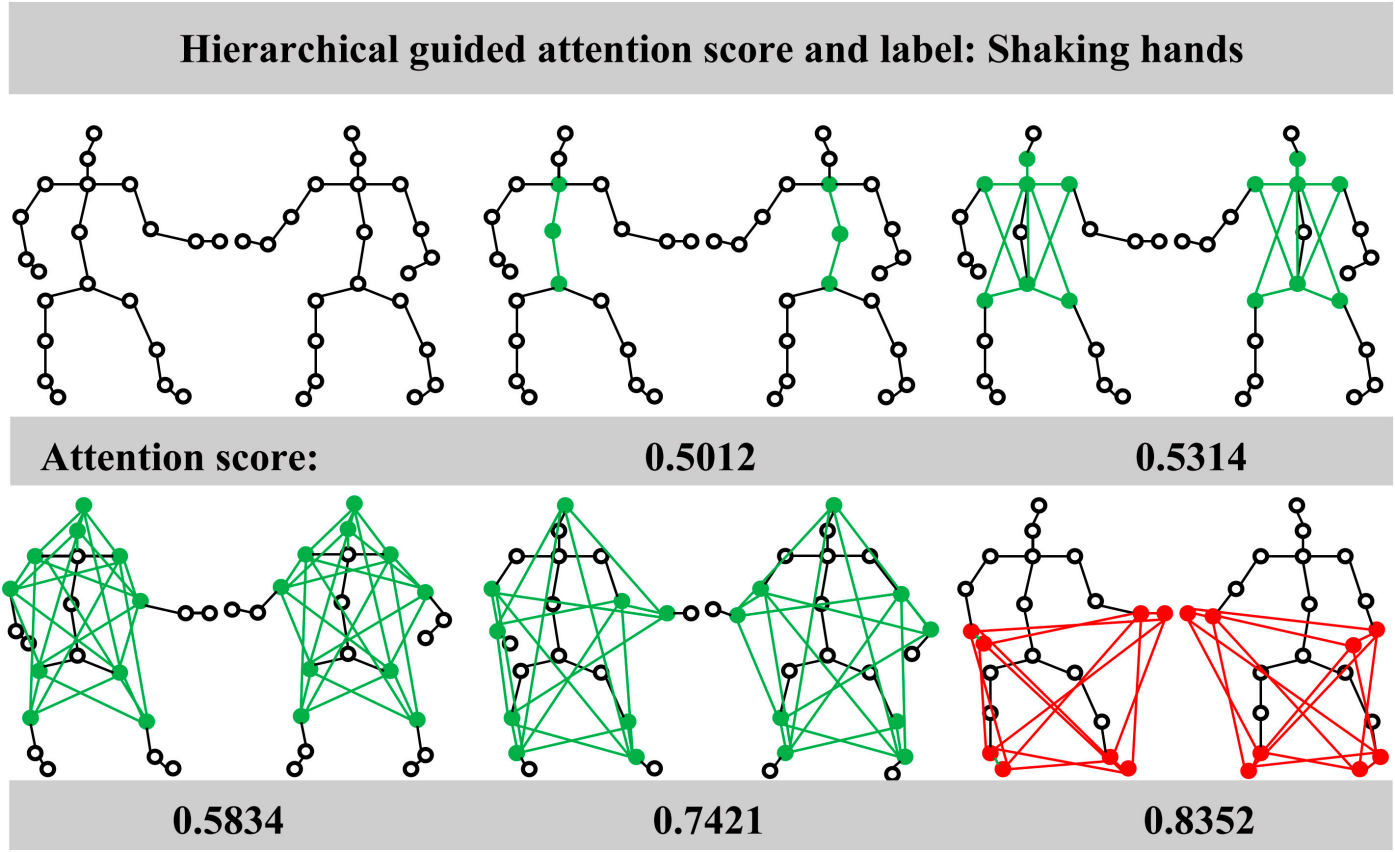
Hierarchical attention scores of the HGA module for the "shaking hands" class.

Next, we validated the effectiveness of the STD module and HGA module on a Merge-and-Split Graph. As shown in Table 1, the STD module improved the accuracy by 0.48%, and the overall network model achieved an accuracy improvement of 1.49%. This strongly demonstrates the importance of extracting short-term spatiotemporal information to better learn subtle differences between similar actions. The HGA module improves the accuracy of the Merge-and-Split Graph by 0.51% and the whole model by 1.52%. For different actions, the information hierarchy that we need to pay attention to is different. The HGA module allocates adaptive weights to each set of hierarchical sets, learning the dependence relationship between each hierarchical set. As shown in Fig. 6, for the “Shaking hands” action, the feature information is clustered around the hand, and the set of the hierarchical sets containing hand-related nodes scored significantly higher than the set of levels without hand-related nodes. The HGA module can highlight the hierarchical set that is more relevant to interaction behavior. It can be found that both our STD and HGA modules are effective.

Finally, we integrated the Merge-and-Split Graph, the STD module, and the HGA module into the network and achieved a significant improvement in accuracy by 1.92% compared to the baseline model. This result confirms the effectiveness of the MS-GCN model in capturing spatiotemporal dependencies and learning hierarchical relationships between actions.

### Comparison with skeleton-based action recognition

**Fig. 7.**
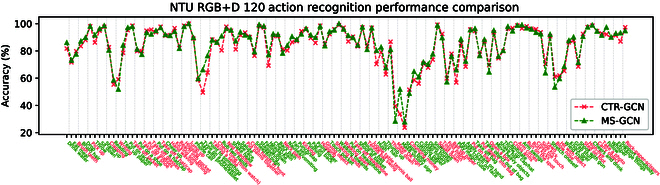
Accuracy comparison of MS-GCN and CTR-GCN on the X-Set of NTU-RGB+D 120 dataset for each of 120 classes. The font color marked pink means that the accuracy of CTR-GCN in this category of action recognition is higher than that of our model, and green means that the accuracy of our MS-GCN in this category of action recognition is higher than that of CTR-GCN.

Although the main task of MS-GCN is human interaction recognition, we also briefly validate the performance of our model on the X-Sub of NTU-120 datasets, which contain not only 2-person interactions but also single-person actions. In CTR-GCN, when dealing with interaction recognition, a common approach involves simplifying the interaction into 2 independent individuals, thereby neglecting the interrelated features between the 2 individuals. In contrast, MS-GCN employs a Merge-and-Split graph which amalgamates dual-person interactions into a unified entity. In the process of single-person recognition, the single person is copied into the form of 2 people in the data preprocessing stage, and MS-GCN is used to extract the features between behaviors. As shown in Fig. 7, the comparison results show that our approach achieves higher accuracy than CTR-GCN in 71 out of 120 action classes, especially for the recognition of interaction behaviors. For instance, the accuracy of “taking a photo” and “following” is significantly higher than CTR-GCN. Moreover, our approach also shows significant advantages in single-person action recognition, such as “type on a keyboard”, “play with phone”, and “make victory sign”, which achieve higher accuracy than CTR-GCN. Our MS-GCN can effectively learn the relevant features of interaction behaviors. Overall, our approach demonstrates superior performance compared to CTR-GCN on the entire NTU120 dataset, which confirms the effectiveness of our proposed MS-GCN.

### Comparison with state-of-the-art methods

Many state-of-the-art methods employ a multistream fusion framework. We adopt the same framework as [50,56] for a fair comparison. Specifically, we fuse the results of 2 modalities, i.e., joint, bone.

The experimental results of interaction categories on the NTU-RGB+D and NTU-RGB+D 120 datasets are shown in Table 2. The proposed MS-GCN outperforms other human skeleton-based interaction recognition methods. Benefiting from the proposed MSGC, STD, and HGA modules, MS-GCN achieves better performance than CNN and RNN-based methods. In the joint modality, MS-GCN has the highest interaction recognition accuracy with 1.38% and 1.56%improvement over CTR-GCN on X-sub and X-set of NTU-RGB+D. On the X-sub and X-set of NTU-RGB+ d120, MS-GCN’s performance improved by 1.89% and 1.67% over CTR-GCN. In the case of 2-stream fusion, the fusion results of joint modality and bone modality, compared with 2s-AIGCN, the performance of MS-GCN is improved by 1.84% and 0.86% on X-sub and X-set of NTU-RGB+D, and is improved by 1.12% and 1.87% on X-sub and X-set of NTU-RGB+ D120. The results show that the proposed method can better express the interaction characteristics of 2 skeletons in time and space.

**Table 2.** Comparisons of interaction recognition performance on NTU-60* and NTU-120*

Method	NTU-60* (%)		NTU-120* (%)	
	X-Sub	X-View	X-Sub	X-Set
FSNET [57] [2019, PAMI]	74.0	80.5	61.2	69.7
ST-LSTM [2] [2016, ECCV]	83.0	87.3	63.0	66.6
ST-GCN [23] [2018, AAAI]	83.3	87.1	78.9	76.1
GCA [1] [2017, CVPR]	85.9	89.0	70.6	73.7
2s-GCA [3] [2017, TIP]	87.2	89.9	73.0	73.3
AS-GCN [36] [2019, CVPR]	89.3	93.0	82.9	83.7
ViT [58] [2020, arXiv]	89.7	92.5	81.5	82.5
LSTM-IRN [35] [2021, ICCV]	90.5	83.5	77.7	79.6
IGFormer [5] [2022, ECCV]	93.6	96.5	85.4	86.5
CTR − GCN_(*joint*)_ [39] [2021, ICCV]	94.3	96.1	87.7	89.0
CTR-GCN [39] [2021, ICCV]	95.31	97.60	92.03	92.82
2s-DRAGCN [56] [2021, Pattern Recognit]	94.68	97.19	90.56	90.43
2s-AIGCN [50] [2022, ICME]	95.34	98.00	90.71	90.65
MS − GCN_(*joint*)_	95.68	97.66	89.59	90.67
MS − GCN_(*joint*, *bone*)_	97.18	98.86	91.83	92.52

## Conclusions

In this paper, we propose a Merge-and-Split Convolutional Network (MS-GCN), which consists of 3 modules: STD, Merge-and-Split Convolution (MSGC), and HGA. In addition, for interaction recognition, we propose the Merge-and-Split Graph to capture and learn interaction information. A large number of experimental results show that the proposed model has better performance on NTU-60 and NTU-120 interaction datasets. The model effectively captures spatiotemporal dependencies and hierarchical relationships between actions, leading to improved accuracy in interaction recognition. There are 2 limitations of this paper. First, for complex motion behaviors, MS-GCN will extract more important correlation features for complex behavior features. Secondly, MS-GCN can only be used for single and 2-person interaction recognition at this stage, and it needs to be further improved to extend to multiperson sports so that it is not limited to interaction recognition. In future work, we will study and improve these limitations.

## Data Availability

Data are available upon reasonable request.
